# A Fully Integrated Sensor SoC with Digital Calibration Hardware and Wireless Transceiver at 2.4 GHz

**DOI:** 10.3390/s130506775

**Published:** 2013-05-21

**Authors:** Dong-Sun Kim, Sung-Joon Jang, Tae-Ho Hwang

**Affiliations:** The Department of Multimedia-IP Research Center, Korea Electronics Technology Institute, #68 Yatap-dong Seongnam-si Bundang-gu, Gyeonggi-do 463-816, Korea; E-Mails: croh@keti.re.kr (S.-J.J.); taeo.hwang@gmail.com (T.-H.H.)

**Keywords:** system-on-a-chip, IEEE 802.15.4, wireless PAN, sensor calibration, Internet of Things

## Abstract

A single-chip sensor system-on-a-chip (SoC) that implements radio for 2.4 GHz, complete digital baseband physical layer (PHY), 10-bit sigma-delta analog-to-digital converter and dedicated sensor calibration hardware for industrial sensing systems has been proposed and integrated in a 0.18-*μ*m CMOS technology. The transceiver's building block includes a low-noise amplifier, mixer, channel filter, receiver signal-strength indicator, frequency synthesizer, voltage-controlled oscillator, and power amplifier. In addition, the digital building block consists of offset quadrature phase-shift keying (OQPSK) modulation, demodulation, carrier frequency offset compensation, auto-gain control, digital MAC function, sensor calibration hardware and embedded 8-bit microcontroller. The digital MAC function supports cyclic redundancy check (CRC), inter-symbol timing check, MAC frame control, and automatic retransmission. The embedded sensor signal processing block consists of calibration coefficient calculator, sensing data calibration mapper and sigma-delta analog-to-digital converter with digital decimation filter. The sensitivity of the overall receiver and the error vector magnitude (EVM) of the overall transmitter are −99 dBm and 18.14%, respectively. The proposed calibration scheme has a reduction of errors by about 45.4% compared with the improved progressive polynomial calibration (PPC) method and the maximum current consumption of the SoC is 16 mA.

## 1. Introduction

During the past several years, in the area of wireless telecommunications a novel paradigm named “the Internet of Things” (IoT), which was first used by Kevin Ashton in a presentation in 1998, has gained more and more attention in academia and industry [1]. The term of IoT refers to the possibility of connecting sensors, actuators or any device to the internet by embedding computational capabilities in all kinds of objects. It will be possible to provide a qualitative and quantitative leap in several sectors: healthcare, logistics, industrial sensing systems, entertainment, and so on. In particular, one of the most important elements in the IoT paradigm is wireless sensor networks (WSN) and sensoring system because the benefits of connecting IoT elements can go beyond remote access only by heterogeneous information systems [2,3]. For wireless connection among devices, IEEE 802.15.4 standard with 6LowPAN (IPv6 over Low-Power Wireless Personal Area Networks) is traditionally focused for its practical considerations of low power, reliability, and security for various cost-sensitive applications. According to many market analysis reports, the industry and home networking sector will be the first to adopt IEEE 802.15.4 devices and the market for this new device is set for explosive growth in less than three years for its simple and improved interoperability.

Along with the rapid development of IEEE 802.15.4 technology, it has been widely tried to use temperature sensors, bio-signals sensors, etc., in a variety of applications for event detection and data gathering. Mapping sensor signals in WSN to physical readings demands a wide range of specific analog signal processing blocks, such as sensor signal amplifiers, analog-to-digital converters (ADC), filters and sensor calibration modules [4]. Therefore, most IoT devices based on WSN separately use analog front-end integrated chips (ICs) to interface with specific sensors, a wireless transceiver system-on-a-chip (SoC) and a tiny embedded microcontroller. Such discrete systems show the limitations of building small form factor solution and low power consumption in various IoT applications. In this reason, challenges to develop fully integrated wireless solutions in silicon-based substrates for sensor system are recently focused on to overcome the problem and meet the demand for small size applications [4,5].

In this paper, we present the development of fully integrated 0.18-*μ*m CMOS sensor SoC with radio transceiver targeted towards wireless sensor networks in 2.4-GHz ISM-band and a sensor signal processor. The remarkable physical layer characteristics of IEEE 802.15.4 at 2.4-GHz are that the radio operates with the data rate of 250 kb/s based on direct sequence spread spectrum (DSSS) using the offset quadrature phase-shift keying (OQPSK) modulation scheme [6–8]. DSSS uses a 32-chip pseudo noise (PN) code and occupies 16 channels each having 5-MHz in the 2.4-GHz [9–12]. For efficient adoption to wireless sensor network, one of our design focus is the monolithic integration with radio and digital circuitry such as digital baseband physical layer (PHY), media access control (MAC) functions and microcontroller. MAC functions support automatic MAC frame generation, cyclic redundancy check (CRC), and inter-symbol timing check. The second design focus is the sensor signal interface circuit that integrates a programmable gain amplifier (PGA), an 10-bit Σ-Δ ADC and a sensor signal calibration processor (SSCP). The rest of this paper is organized as follows. Section 2 describes the system architecture. The implementation of the fully integrated RF transceiver and sensor signal processor is presented in Section 3. Section 4 shows the measurement results and the conclusions are presented in Section 5.

## 2. System Architecture

Ultra-miniature and low power sensor platforms for IoT application essentially need the high level of integration and functional flexibility. Radio frequency (RF) modules for data transmission and the sensor interface circuitry for signal processing are key components in the whole system because low-cost smart sensor systems increasingly contain multiple sensors of different types, which require heterogeneous interfaces for pre-amplification, pre-filtering, sensor data calibrating and reading [13]. Integrating a programmable processor and an adaptive sensor interface into one SoC combines analog and digital processing capability and permits rapid customization to different applications using the same integrated circuit. In addition, single-chip integration of these components enhances performance while simultaneously reducing size, cost and power consumption [14].

**Figure 1.**
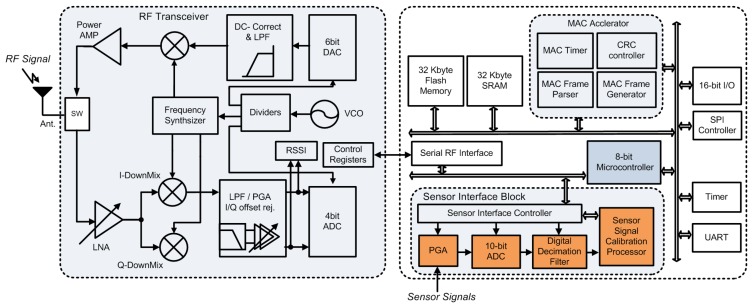
Architecture of a fully integrated sensor SoC.

The proposed system architecture, which includes both analog sensor signal acquisition front-end, digital signal interface block for self-calibration without the need for external calibration by the user and IEEE 802.15.4 compliant wireless transceiver, is shown in Figure 1. The analog front-end consists of chopper-stabilized preamplifier followed by variable gain switched capacitor type amplifier with adjustable gain and bandwidth. Fully-differential architecture is employed in order to suppress the common-mode noise and interferences on the signal. For interfacing between the analog front-end and digital sensor signal calibration processor (SSCP), a second-order 10-bit sigma-delta ADC with digital decimation filter follows the front-end to convert the amplified signal to digital format. Most analog sensors have own non-linearities and these properties vary with external temperature. A proposed SSCP consists of genetic algorithm based polynomial optimization block and can calculate coefficients of calibration polynomial without external computation modules such as personal computers (PCs).

For collecting environmental data such as temperature, pressure, position, flow, humidity, vibration, force and motion to monitor the real-world, 2.4 GHz IEEE 802.15.4 wireless transceiver is fully integrated and it includes a receiver, transmitter, frequency synthesizer with on-chip voltage-controlled oscillator (VCO), 6-bit I/Q DACs, 4-bit I/Q ADCs, and digital MAC functions. The receiver adopts zero-IF architecture to have low power consumption, low cost and small size [15–17]. The 2.4-GHz RF signal is first amplified by a low noise amplifier (LNA) and then down-converted to zero-IF I/Q signals by two identical mixers driven by quadrature LO signals from a frequency synthesizer. At the analog baseband stage, a third-order RC filter and a programmable gain amplifier are used simultaneously to perform channel selection filtering, signal amplification, and DC-offset cancellation. The power detector of digital baseband finds the beginning of the received signal and calculates the average power value for link quality indication (LQI). The CFO compensation block also remove the frequency offset error and the symbol timing synchronization block recovers symbol timing to acquire exact chip sequence. After the above signal recovery processing, the signals are de-spreaded and decoded to decide payload data. The transmitter adopts a zero-IF modulation with up-conversion mixer using a current mixing scheme. The baseband OQPSK signals generated by digital modulator are followed by 6-bit I/Q DAC. A mixer directly up-converts the baseband signal to 2.4-GHz, which is combined by RC low-pass filter. Since OQPSK modulation is a constant envelop modulation, a nonlinear power amplifier with high efficiency can be used for high power emission. For generating 2.4-GHz LO signals with 5-MHz channel spacing, a delta-sigma fractional-*N* frequency synthesizer derived from a 16-MHz crystal oscillator is implemented. The 2.4 GHz LO signals are generated by a complementary differential voltage controlled quadrature oscillator (QVCO) with a small area and high *Q* on-chip inductor. The frequency synthesizer is implemented in fully differential type, for immunity to common mode noise.

To reduce the processing power of digital baseband, we focus on the design of hardware MAC and it supports CRC, frame generation, auto retransmission, and inter-symbol time violation check. In addition, a conventional 8051 architecture based 8-bit microprocessor with a simple 8-bit instruction set is integrated to perform the processing of raw sensor data and control internal function blocks such as a SSCP and wireless transceiver with digital baseband. The 8-bit address and data buses provide on-chip interconnection between the SSCP and other functional blocks including on-chip SRAM, flash memory, clock management unit, UART, SPI, and 16-bit GPIOs. The motivation of employing SSCP and configurable PGA with 10-bit sigma-delta ADC in sensor interface block is to provide flexible configuration of the various sensor systems for tens of different types of sensors. On-chip SRAM provides buffering for data calculations as well as program code storage, and UART offers the communication interface between the SoC with external devices or host personal computer (PC) for data post processing.

## 3. Implementation

The design flow employed in the proposed SoC consists of standard synthesis, placement and route flow for digital blocks, as well as full custom design flow for analog and RF mixed-signal blocks. Combining different design flows as such has a challenge in verifying the whole mixed system with good coverage and fast simulation speed. Due to the design tool resource limit, full chip-level SoC verification before sign-off cannot be generally performed. Alternatively, digital and analog sub-systems are verified at the layout-level separately with their specific stimuli vectors such as captured RF signals and versatile sensor output data before the final SoC integration is performed in this design [5]. The digital block mainly consists of digital baseband, 8-bit microcontroller, MAC functions and sensor signal interface block. An embedded microcontroller performs software MAC, sensor network protocols and sensor application softwares. It operates up to maximum 30 million instructions per second (MIPS) and provides programmable general purpose input output (GPIO). It also supports 64 Kb programmable ROM and 32 Kb SRAM. The program codes in ROM area are copied to SRAM for fast execution, and internal control registers for digital transceiver and sensor signal interface can be accessed by address from 0xFF00 to 0xFFFF.

### 3.1. RF Transceiver

**Figure 2.**
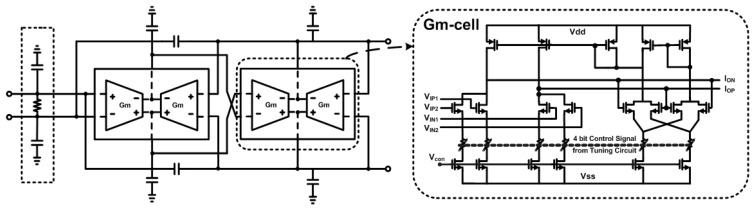
Channel selection filter with third-order Butterworth LPF.

**Figure 3.**
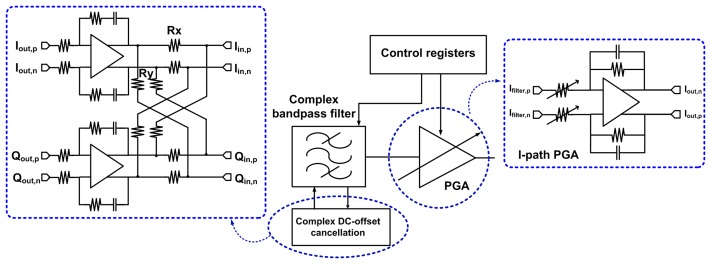
Low-IF Receiver Architecture.

A RF transceiver is designed using 0.18-*μ*m mixed-signal CMOS process to include six metal layers with 2-*μ*m thick top metal. This process provides high gain and good quality factor, *Q* for on-chip inductor, resulting in low power consumption in RF and analog circuits. The RFE consists of low-noise amplifier (LNA) and quadrature down-conversion mixer. The fully balanced 2.4-GHz LNA uses current-reuse complementary technique without inductor for preventing large area and uses a common source with resistive feedback. Input matching circuit is realized by external passive LC components. The LNA features 6.87 dB noise figure (NF) and a third-order input intercept point (IIP3) of −12 dBm at maximum gain. The differential outputs of LNA are directly down-converted into a common analog baseband path by a Gilbert-cell-based quadrature frequency demodulator. The selection of the vertical bipolar transistors in the switching quadrant decrease the gain of mixer; however, the average integrated noise floor in the direct-conversion receiver improves due to the reduced 1/*f* noise. The channel selection low pass filters are realized as balanced Gm-C biquadratic filters to achieve a low current consumption. High linearity and a constant wide bandwidth are achieved by using a new transconductance (Gm) cell. The third order Butterworth filter is implemented by cascading a biquad cell and a single pole cell. The programmable gain cell is stationed at the middle to improve the cascaded dynamic range. For filter requirements, 1/*f* noise should be reduced and the DC offset should be small because of its direct conversion receiver (DCR) structure [18,19]. In addition, most of the interference is also filtered in the first part to alleviate the spurious free dynamic range (SFDR) requirements of the PGA and the ADC. Figure 2 shows the designed third Butterworth LPF and the proposed Gm-cell with degeneration resistor. Two Gm-cells are used as one to reduce the area that LPF occupies. Because the signal level of the RF input requires a minimum dynamic range of 81 dB from −108 dBm to −20 dBm, the PGA utilizes the three gain stages to control the gain of 0∼73 dB by a 1 dB step as shown in Figure 3.

**Figure 4.**
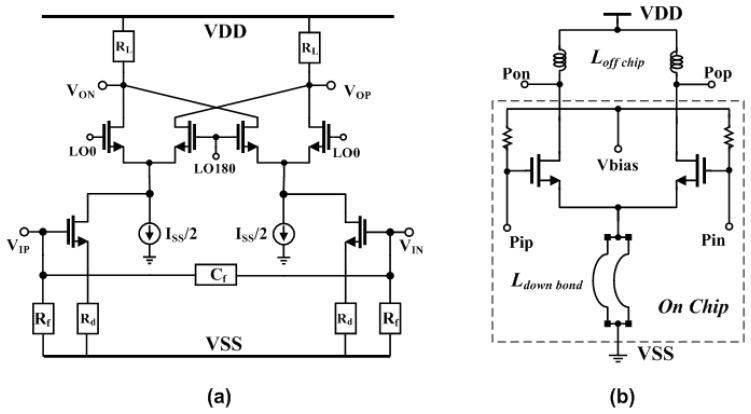
Transmitter circuits. (**a**) Up-conversion I/Q-modulator using current-mixing scheme. (**b**) Drive-amplifier with off-chip inductor.

The resistor switching method is utilized in order not to lose the linearity of PGA, and 8-bit digital data for I/Q from the baseband are input to control the DC offset at the back side of PGA1 to use the feedback loop to reduce the offset at the LPF output for DC offset cancelation. The DC offset change at the LPF output is ±130 mV and I/Q 4bit dual flash-ADCs are designed for interface of baseband block. In the transmitter path, OQPSK modulated baseband signal is converted from digital to analog before being applied to frequency up-translation block. Figure 4(a) shows the circuit of up-conversion mixer with RC low-pass filter. The baseband analog signal is filtered by second RC low-pass filter and it is translated into RF frequency by up-conversion modulator with balanced Gilbert-cell using current-mixing scheme. The major advantage of current mixing relaxes a requirement of heavy linearity of modulator inputs from high voltage-driving DAC output signal. In addition, this scheme for frequency-up modulation can produce satisfactory results for high modulation quality, low-power consumption, and good linearity This balanced mixer converts baseband signal directly up to 2.4 GHz and delivers −20 dBm differential signal to power amplifier. LO emission is due to differential mismatch in the modulator circuit, while spectrum re-growth is due to LO (0/90-degree) quadrature imbalance and nonlinearity of the Gilbert-cell. Layout is fulfilled very carefully to maintain symmetry for differential and quadrature signals, which minimizes both LO emission and spectrum re-growth. Figure 4(b) shows the driver amplifier of a differential common source topology with off-chip inductor having a high Q.

**Figure 5.**
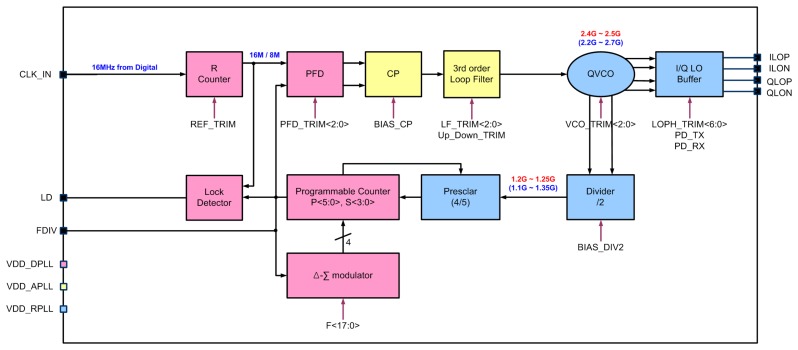
Frequency synthesizer block-diagram.

The fractional-*N* frequency synthesizer with a third-order passive loop filter generates the LO signal for transmit/receive mode. A crystal reference of 16 MHz is internally divided and the 2.4 GHz LO signals are generated by complementary differential voltage controlled quadrature oscillator (QVCO) as shown in Figure 5. The LC-resonator consists of four-turn spiral inductor and varactor. The tuning frequency of QVCO is simulated from 2.127 GHz to 2.756 GHz. The divider circuit for high frequency has a structure of negative-feedback type using two latches. The phase frequency detector (PFD) consists of two D-flip-flop (DFF), AND-gate, and delay-time block for locking speed and high linearity of phase transfer function. The charge-pump circuit has a structure of nMOS/pMOS cascade-type to minimize up/down current mismatch and output switching noise. The clock generation block provides a reference clock of PLL and sampling-clocks of ADC/DAC using an external 16-MHz crystal-oscillator.

### 3.2. Digital Baseband with Hardwired MAC Functions

Digital baseband is made up of OQPSK DSSS modulator and demodulator for transmitting or receiving the radio frequency signals. The data bit stream is coded into pre-defined 4-bit symbols where each of the 16 symbols consists of a nearly orthogonal 32-chip pseudo-random sequence. The overall data bit to symbol mapping effectively implements a DSSS scheme with a chip rate of 2 Mchips/s from a data rate of 250 kbps. The signal spreading and O-QPSK modulation with half-sine pulse shaping are performed digitally. The modulated and spread I/Q baseband signals are applied to the digital-to-analog converters, whose outputs are low-pass-filtered and up-converted directly to RF by a single-sideband modulator [20–22].

**Figure 6.**
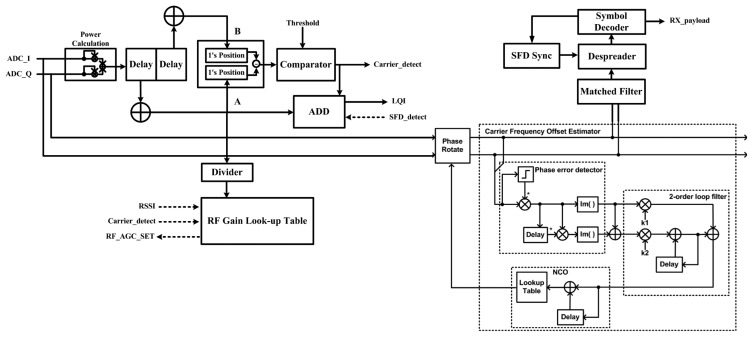
Synchronization block for digital baseband.

As shown in Figure 6, the synchronization for digital baseband consists of 5 blocks: power detector, AGC block, carrier frequency offset (CFO) compensation block, symbol timing synchronization block, and despreading block. Power detector finds the beginning of the received signal using the double windowing method [23] and it also calculates the average power value for link quality indication (LQI) until the start of the frame delimiter (SFD) signal is detected. The CFO compensation block estimates the carrier frequency offset and it contains a phase error detector, loop filter, numeric controlled oscillator (NCO) and phase rotator. The phase of the received signal, *ADC_I* and *ADC_Q*, is compared with NCO signals in a frequency discriminator. The resulting discriminator output is proportional to the frequency offset of the received signal and is fed through a loop filter to NCO, which synthesizes local oscillator signals used to remove the frequency offset error. It can compensate the measured ADC sample data with −4.78 dB receiver input power and recover the distorted signal up to ±50 ppm. The AGC block calculates the average power of one preamble signal after signal detection. It coarsely controls the gain of the amplifier in the RF block using the serial peripheral interface (SPI) and finely trims the output power using the next one preamble signal power. The symbol timing synchronization block recovers symbol timing to acquire the exact chip sequence and it is implemented using a non-decision directed techniques based on Gardner's algorithm [24]. The despreading of DSSS signal is performed by matched filter correlators to decide the received chip sequences [23,25], and the closest fitting sequence is selected using maximum likelihood techniques.

**Figure 7.**
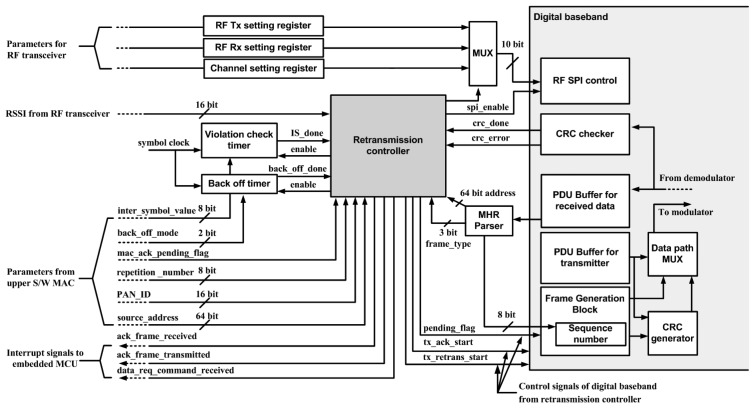
Block diagram of digital MAC function block.

For fast and low power execution of MAC protocols, digital MAC functions support CRC, frame generation and violation check timer such as acknowledgement response time are implemented as shown in Figure 7. The frame generation block organizes the transmitted data frame and parses the information from the received frames. It also provides the operation of automatic transmission of the acknowledgement, data frame retransmission, and channel scanning for carrier sense multiple access-collision avoidance (CSMA-CA) protocols. All digital MAC functions communicate with embedded microcontroller by interrupt signals and control registers. The data path multiplex (MUX) block controls the direct data flow between digital MAC blocks without microcontroller data processing.

### 3.3. Sensor Signal Interface Block

**Figure 8.**
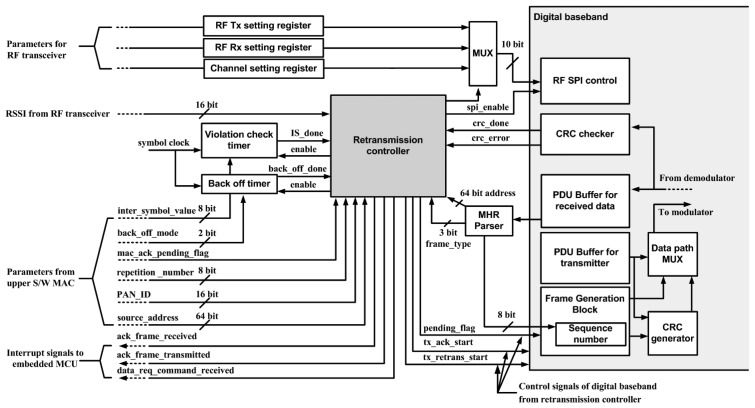
Block diagram of digital MAC function block.

Many current applications for sensor systems require a combination of different sensors to properly interface to the broad diversity of real world phenomena [26], and the development of intelligent sensors with readjustment capabilities is imperative in order to facilitate calibration while not increasing its costs [27]. Several generic sensor interface circuits have been introduced for these applications and these circuits support an extensive variety of sensors at the cost of hardware efficiency [28–31]. However, they do not provide the signal processing capabilities necessary to decentralize network workloads or provide an embedded sensor calibration hardware function. This paper presents a hardwired configurable sensor interface, which supports programmable gain adjustment, 10-bit ADC with digital decimation filter, and sensor signal calibration processor for the improvement of a progressive polynomial algorithm that facilitates the calibration process due to the self-adjustment of sensor. To achieve low power consumption and small system size with a moderate system speed, a Σ-Δ architecture that features low power, low hardware complexity and good robustness is chosen for the analog front end (AFE) as shown in Figure 8. For programmable gain amplification, a fully differential resistive feedback topology is adopted and the closed loop voltage gain is given by

(1) $${\text{Gain}}_{\text{overall}}=\left[1+\frac{R_{2}}{R_{1}}\right]\text{PGA}1\times \left[\frac{R_{2}}{R_{1}}\right]\text{PGA}2$$

The main architecture of PGA consists of two gain stages and an output buffer stage with 0 dB gain for driving the following analog-to-digital converter. Gain tuning is accomplished by switching feedback resistors with a 5-bit decoder. The first stage can realize a gain range of 2 to 16, while the second stage achieves discrete gain tuning steps ranging from 0.5 to 64. Owing to proper combination of gain switching, an overall gain range of 0 dB to 18 dB with 2 dB per step is obtained with appropriate decoding. A second-order 1.5 bit Σ-Δ modulator with capacitor sharing technique and three-level 1.5 bit quantizer is utilized in this design [32,33]. The modulator samples the input at a high rate for producing a binary output whose average value over time tracks the analog input, and it consists of a loop-filter that has a transfer function and a digital-to-analog converter (DAC) in the feedback path. The Σ-Δ ADC design achieves the maximum sampling rate of 500 KS/s with a maximum 1 MHz over-sampling rate. The input dynamic range (DR) is measured to be 105 dB and the maximum power consumption is determined to be 8 mA with 1.8 V power supply.

We especially focus on the sensor signal calibration processor for overcoming previous microcontroller based sensor signal processing. Most sensors require calibration and linearization before they can be used reliably in a system. Often the microcontroller in the system is used to compute correction curves for each sensor, and each signal sample is passed through a digital correction formula. The improved progressive polynomial calibration (PPC) methods are generally used to calculate correction curves because of its excellent accuracy [34,35]. However, this demands system computation power and implicates a much slower signal processing, especially in systems with multiple sensors. In addition, it is impossible to calculate coefficients for digital correction formula in an embedded processor. This problem is often tackled by using high-speed DSP, and coefficients of digital correction formula are independently calculated by host personal computers.

**Figure 9.**
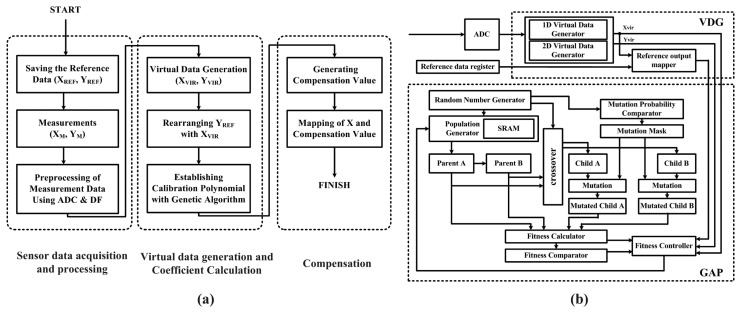
(**a**) Proposed sensor correction flow (**b**) Block diagram of sensor signal calibration Processor.

In this paper, we propose a digital sensor signal processor with the virtual data generation (VDG) that can cover all ranges with the small measurement data for sensor calibration. We combine the VDG with genetic algorithm (GA) to fill the gap between a real sensor characteristic and virtually generated data and to establish the optimal calibration polynomial quickly The reference data and measurement data are acquired and have to be prepared with processing ADC with decimation filter in advance for the VDG operation. The VDG generates the virtual data between the measurement data and rearranges the reference output according to the generated virtual sensor input. At the final coefficients calculation process for the correction formula, the GA finds the optimal coefficients of a polynomial with them and it tries to fit the polynomial to match the real sensor errors all over the range plus the generated virtual CPs. After establishing a calibration polynomial, a compensation according to a sensor input is available as shown in Figure 9(a).

To generate the virtual data, the VDG uses a linear interpolation in case of the 1-dimensional (1D) calibration, which considers the effects by only sensor inputs, and a bilinear interpolation is used for the 2-dimensional (2D) calibration with both sensor inputs and the environmental variables. These interpolations are used for the rearranging the reference output with virtual sensor inputs, and the 2D operation of VDG, *p*_2_*_D_*(*x*, *t*) can be denned as below.

(2) $$p_{1D}(x,t)=\sum_{n=1}^{N-1}\sum_{m=1}^{M-1}\left\{f(x_{m},t_{n})+\sum_{k=0}^{N_{1}}\frac{k}{N_{1}+1}\left(\frac{f(x_{m+1},t_{n})-f(x_{m},t_{n})}{x_{m+1}-x_{m}}\right)\right\}$$

(3) $$p_{2D}(x,t)=\sum_{m=1}^{(N_{1}X(M-1)+M)-1}\sum_{n=1}^{N-1}\left\{p_{1D}(x_{m},t_{n})+\sum_{k=0}^{N_{2}}\frac{k}{N_{2}+1}\left(\frac{p_{1D}(x_{m},t_{n+1})-p_{1D}(x_{m},t_{n})}{t_{n+1}-t_{n}}\right)\right\}$$

where *x* and *t* denote the sensor input and the environmental variable, respectively, *M* is number of *x*, *N* is number of *t*, *N*_1_ is number of the virtual data between each *x*, *N*_2_ is number of the virtual data between each *t*, *f* is sensor output, *p*_1_*_D_* is a linear interpolation in the *x*-direction and *p*_2_*_D_* is a linear interpolation in the *t*-direction. After finishing the generation of virtual data and rearranging of reference data by the VDG, the GA starts to find its optimum coefficients. A general operation of GA consists of the randomly multiple populations generation, the natural selection of two parent populations, the generation of two child populations by the crossover between two selected parents and the mutation, the fitness calculation of them, and the replacement of a parent with a child after the fitness comparison. We map the coefficients of the polynomial to the chromosomes in the population. In addition, the fitness can be expressed as the inverse function of the difference between the real sensor error and the polynomial output according to inputs and each coefficient. The GA performs repeatedly until getting target fitness and the coefficients are changed to match real sensor error by the crossover and mutation [36]. Figure 9(b) shows the overall structure, which consists of the virtual data generation unit (VDGU) and the genetic algorithm processor (GAP). The VDGU is implemented with the linear and bilinear interpolator. A random number generator block generates the initial population and denotes the crossover cut point on the parents and the mutation inverted mask point. The mutation probability comparator block generates a mutation mask and consists of a hamming distance calculator, 5 × 5 fixed point multiplier, 10/5 divider and exponential function calculator. A hamming distance calculator is simply composed of XNOR gate and the exponential function calculator is implemented based on lookup table. Mutation block simply performs XOR operation with mutation mask and crossover results [36,37].

## 4. Measurement Results and Discussion

**Figure 10.**
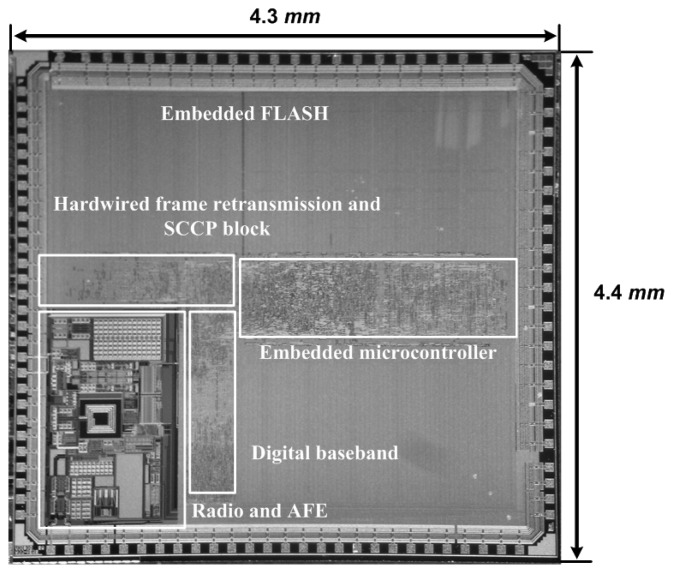
Die microphotograph.

A sensor SoC die microphotograph, which consists of IEEE 802.15.4 2.4 GHz transceiver, digital baseband with MAC functions, and sensor signal interface block, is shown in Figure 10. The total die area is 4.3 × 4.4 *mm*^2^ and it consumes a maximum of 29 mW, and a LPCC-48 package is used.

**Figure 11.**
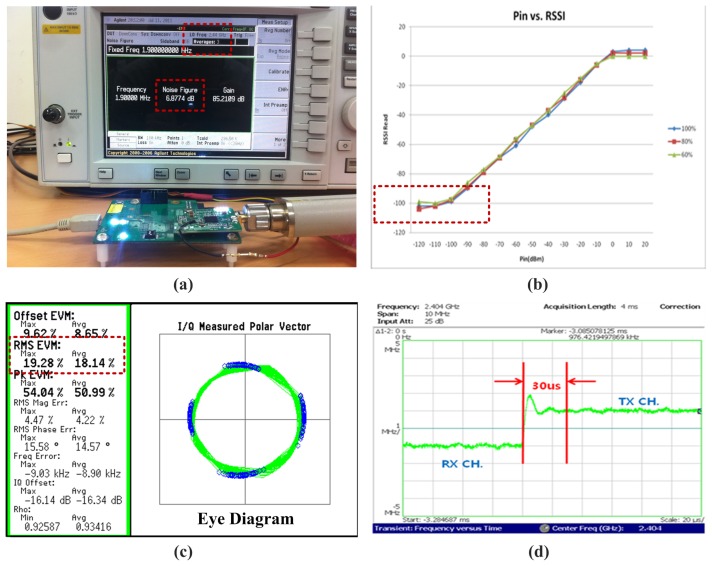
IEEE 802.15.4 RF Transceiver Measurement Results. (**a**) Receiver cascaded noise figure (NF); (**b**) The maximum gain of AGC; (**c**) Vector signal analysis of transmitter; (**d**) PLL turnaround time.

As shown in Figure 11(a), RF signal characteristics are measured by Agilent IEEE 802.15.4 measurement kits and the measured overall cascaded-NF of receiver is 6.87 dB for 2.4 GHz band for IEEE 802.15.4 OQPSK mode. Measurement results show that the overall receive cascaded-IIP3 is −12 dBm and the maximum gain of the receiver is about 100 dB as shown in Figure 11(b). The automatic gain control (AGC) of the receiver is 96 dB with 1 dB step and the selectivity is −15 dBm. Because of the low in-band integrated phase noise and the digital calibration that eliminates I/Q mismatch and baseband filter mismatch, transmitter EVM is dominated by nonlinearities. As shown in Figure 11(c,d), a reference design achieves 18.14% EVM for an output power of 0 dBm and the turnaround time of PLL that change operation mode from receiver to transmitter is 30 *μs*. In order to measure the power consumption of the SoC's communication module, a National Instrument PXI4071 multi-meter device was used, with a transmit data length of 20 bytes, a supply voltage of 3.3 V, and an MCU clock rate of 32 MHz. Table 1 provides descriptions of two power management modes and the average amount of current consumed in each mode. In the proposed power management mode, the embedded MCU and peripherals are disabled, but the RF transceiver, digital baseband with hardwired MAC digital demodulator block and sensor interface block are active for transmitting acquired sensor data. It shows that the proposed power management method improves the average power efficiency about 18.5%.

**Figure 12.**
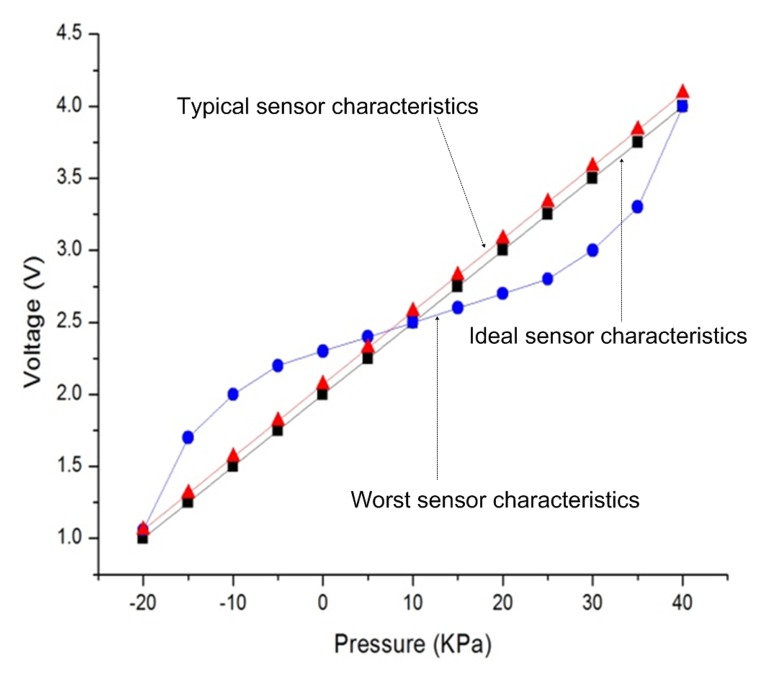
Test data for SSCP performance measurement.

**Figure 13.**
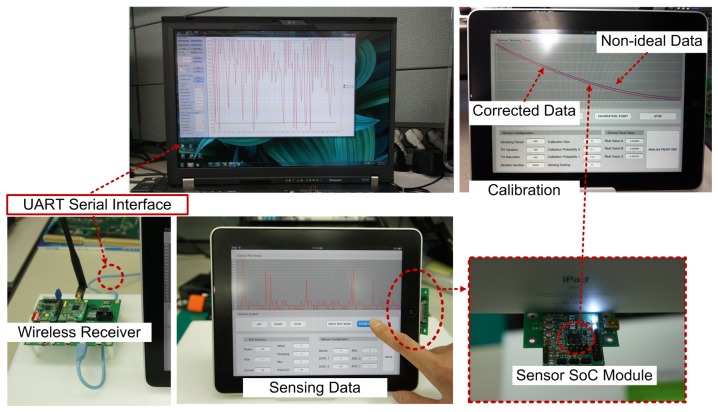
Verification with prototype sensor platform.

For estimating the performance of the SSCP block, the characteristics of a pressure sensor (ADPW) (Panasonic Corporation PS-A(ADPW) pressure sensor: Tokyo, Japan) are used to perform the experiments. We define two kinds of the measurement data for the experiments as shown in Figure 12. The first curve type has ±10% nonlinearity for the worst case, and the other case is a straight line type with 0.06 V offset and 0.01% gain error for the typical case. Parameters for the GAP are population number, chromosome length, crossover probability, crossover type and mutation probability. These parameters are normally configured to 64, 20 bit, 1, 2-point and 0.5, respectively. Table 2 shows the performance with four CPs. The errors of the worst case are reduced by 96.84% with a 3-order polynomial. In the typical case, the remaining errors are almost zero with only three CPs and 2-order polynomial. The maximum computation time is measured to be 25.43 *ms* with 22 MHz clock speed. We also compare the modified PPC [35] with the worst test case. Table 3 shows the comparison results in terms of degree, CPs, and errors. The proposed scheme has more reduction of errors by about 70% with the same four CPs, and the modified PPC with six CPs shows abou 2 times more errors than the proposed SSCP with four CPs. Finally, we implement a test sensor platform for verification with applications as shown in Figure 13. Fully integrated sensor SoC collects sensor data from an embedded ADC with SSCP. It sends corrected data to commercial mobile device by UART serial communication port and transmits the same data by an embedded wireless transmitter. Transmitted sensing data is received by another sensor node and also transferred to a personal computer by UART serial communication port.

## 5. Conclusions

A fully CMOS integrated sensor SoC with a 2.4 GHz wireless transceiver for IoT applications is implemented and measured. The SoC is fabricated in 0.18 *μ*m mixed-signal CMOS process and packaged in LPCC-48 package. It consists of a receiver, a transmitter, digital baseband with hardware MAC accelerator, 8-bit microcontroller and a sensor signal processor with on-chip ADC with PGA. Digitally implemented sensor signal processing functions reduce the processing power of embedded microcontroller and fully support sensor signal calibration and correction without host personal computer or dedicated high performance microcontroller. The overall receiver cascaded noise-figure, sensitivity, and cascade IIP3 are 6.8 dB, −99 dBm, and −12 dBm, respectively. The overall transmitter achieves less than 18.14% error vector magnitude (EVM) for 250 kbps mode. An embedded RF transceiver and baseband hardware fully supports the IEEE 802.15.4 WPAN standard in 2.4-GHz mode. The chip uses 1.8 V power supply and the maximum current consumption is 29 mW for concurrently RF transmission and sensing mode.

## Figures and Tables

**Table 1.** Power consumption Comparison results.

Power Management Mode	Average Current (mA)	Description
Normal Mode	27	All blocks of SoC are active.
Bypass MCU Mode	22	Main processor is disabled.

**Table 2.** Performance results of SSCP.

Test Cases	Degree (order)	CPs number	Errors (%)	Calibration time (ms)
Worst	3	4	3.16	25.43
Typical	2	3	0.001	10.98

**Table 3.** Comparison results with modified PPC.

	Degree (order)	CPs number	Errors (%)

Modified PPC	4	4	10.38
		
	6	6	6.96

Proposed SSCP	3	4	3.16

## References

[1] Yang D.-L., Liu F., Liang Y.-D. A Survey of the Internet of Things.

[2] Santucci G. From Internet of Data to Internet of Things.

[3] Tozlu S., Senel M., Keshavarzian Abtin (2012). Wi-Fi enabled sensors for internet of things: A practical approach. IEEE Commun. Mag..

[4] Fabris E.E., Carro L., Bampi S. A Digitally Reconfigurable Sensor Interface for SOC Using Delta-Sigma Modulators.

[5] Xi J., Yang C., Mason A., Zhong P. (2006). Adaptive multi-sensor interface System-On-Chip. Proc. IEEE.

[6] Zhang C., Wei C., Jiang H., Wang Z. A Baseband Transceiver for Multi-Mode and Multi-Band SoC.

[7] Razov T., Oletic D., Kuri M., Bilas V. MasliNET: A Wireless Sensor Network Based Environmental Monitoring System.

[8] Sun Y., Li L., Luo H. Design of FPGA-Based Multimedia Node for WSN.

[9] Xu W., Yue Y., Zhou T. System Architecture Design of the Internet of Things based on ZigBee.

[10] Healy W.M., Zhou M.C. (2012). Impacts of 2.4-GHz ISM band interference on IEEE 802.15.4 wireless sensor network reliability in buildings. IEEE Trans. Instrum. Meas..

[11] Villarejo M.V., Zapirain B.G., Zorrilla A.M. (2012). A stress sensor based on Galvanic Skin Response (GSR) controlled by ZigBee. Sensors.

[12] Teo T.H., Qian X., Gopalakrishnan P.K., Hwan Y.S., Haridas K., Pang C.Y., Cha H.-K., Je M. (2010). A 700 *μ*W wireless sensor node SoC for continuous real-time health monitoring. IEEE J. Solid-State Circuits.

[13] Boukabache H., Escriba C., Zedek S., Medale D., Rolet S., Fourniols J.Y. (2012). System-on-Chip integration of a new electromechanical impedance calculation method for aircraft structure health monitoring. Sensors.

[14] Liu X., Zheng Y., Phyu M.W., Endru F.N., Navaneethan V., Zhao B. (2012). An ultra-low power ECG acquisition and monitoring ASIC System for WBAN applications. IEEE J. Emerg. Sel. Top. Circ. Syst..

[15] Perumana B.G., Mukhopadhyay R., Chakraborty S., Lee C.-H., Laskar J. (2008). A Low-Power Fully Monolithic Subthreshold CMOS Receiver With Integrated LO Generation for 2.4 GHz Wireless PAN Applications. IEEE J. Solid-State Circuits.

[16] Camus M., Butaye B., Garcia L., Mathilde S., Pellat B., Parra T. (2008). A 5.4 mW/0.07 *mm*^2^ 2.4 GHz Front-End Receiver in 90 nm CMOS for IEEE 802.15.4 WPAN Standard. IEEE J. Solid-State Circuits.

[17] Li Z., Jiang Y., Shu H., Hou N. A 5-GHz Frequency Synthesizer with AFC for Low IF ZigBee Transceiver Applications.

[18] Feng Y., Takemura G., Kawaguchi S., Itoh N., Kinget P.R. (2011). Digitally assisted IIP2 calibration for CMOS direct-conversion receivers. IEEE J. Solid-State Circuits.

[19] Zhou Y., Pan Z. (2011). Impact of LPF mismatch on I/Q imbalance in direct conversion receivers. IEEE Trans. Wirel. Commun..

[20] Gutierrez J.A., Callaway E., Barrett R. (2003). Low-Rate Wireless Personal Area Networks: Enabling Wireless Sensors with IEEE 802.15.4.

[21] IEEE Standard for Part 15.4 (2006). Wireless Medium Access Control (MAC) and Physical Layer (PHY) specifications for Low Rate Wireless Personal Area Networks (LR-WPANs).

[22] Ding G., Sahinoglu Z., Orlik P., Zhang J., Bhargava B. (2004). Broadcasting on IEEE 802.15.4 and ZigBee, Technical Report.

[23] Shengli Z., Giannakis G.B., Ananthram S. (2002). Digital Multi-Carrier Spread Spectrum Versus Direct Sequence Spread Spectrum for Resistance to Jamming and Multipath. IEEE Trans. Commun..

[24] Oerder M. (1987). Derivation of Gardner's timing-error from the maximum likelihood principle. IEEE Trans. Commun..

[25] Elson J., Estrin D. (1965). Time Synchronization for Wireless Sensor Networks.

[26] Yang C., Mason A., Xi J., Zhong P. (2006). Configurable Hardware-efficient interface circuit for multi-sensor microsystems. Proc. IEEE.

[27] Rivera J., Herrera G., Mario C., Acosta P., Carrillo M. (2008). Improved progressive polynomial algorithm for self-adjustment and optimal response in intelligent sensors. Sensors.

[28] Zhang J., Zhou J., Balasundaram P., Mason A. (2003). Configurable hardware-efficient interface circuit for multi-sensor microsystems. Proc. IEEE.

[29] Chao G., Li X., Meijer G.C.M. (2004). A system-level approach for the design of smart sensor interfaces. Proc. IEEE.

[30] Mackensen E., Muller C. (2005). Implementation of reconfigurable micro-sensor interfaces utilizing FPAAs. Proc. IEEE.

[31] Garcia-Ramirez A.G., Osornio-Rios R.A., Granados-Lieberman D., Garcia-Perez A., Romero-Troncoso R.J. (2012). Romero-troncoso smart sensor for online detection of multiple-combined faults in VSD-fed induction motors. Sensors.

[32] Soufi B., Malik S.Q., Geiger R.L. A capacitor sharing technique for RSD cyclic ADC.

[33] Melo J.de. A 3rd Order 1.5-bit Continuous-Time (CT) Sigma-Delta Modulator Optimized for Class D Audio Power Amplifier.

[34] Rivera J., Herrera G., Chacon M. (2009). Improved progressive polynomial algorithm for self-calibration and optimal response in smart sensors. Measurement.

[35] Rahili S., Ghaisari J., Golfar A. (2012). Intelligent selection of calibration points using a modified progressive polynomial method. IEEE Trans. Instrum. Meas..

[36] Kim D.-S., Jeon I.-J., Lee S.-Y., Rhee P.-K., Chung D.-J. (2006). Embedded face recognition based on fast genetic algorithm for intelligent digital photography. IEEE Trans. Consumer Electron..

[37] Kim D.-S., Lee S.-S. (2012). Improved mutation method for providing high genetic diversity of genetic algorithm processor. IEICE Electron. Express.

